# Incidence, prognostic factors, and outcomes of venous thromboembolism in critically ill patients: data from two prospective cohort studies

**DOI:** 10.1186/s13054-021-03457-0

**Published:** 2021-01-12

**Authors:** Ruben J. Eck, Lisa Hulshof, Renske Wiersema, Chris H. L. Thio, Bart Hiemstra, Niels C. Gritters van den Oever, Reinold O. B. Gans, Iwan C. C. van der Horst, Karina Meijer, Frederik Keus

**Affiliations:** 1 Department of Internal Medicine, University Medical Center Groningen, University of Groningen, P.O. Box 30.001, 9700 RB Groningen, The Netherlands; 2 Department of Critical Care, University Medical Center Groningen, University of Groningen, Groningen, The Netherlands; 3 Department of Epidemiology, University Medical Center Groningen, University of Groningen, Groningen, The Netherlands; 4 Department of Anaesthesiology, University Medical Center Groningen, University of Groningen, Groningen, The Netherlands; 5 Department of Critical Care, Treant Zorggroep Emmen, Emmen, The Netherlands; 6 Department of Intensive Care, Maastricht University Medical Center+, Maastricht, The Netherlands; 7 Cardiovascular Research Institute Maastricht (CARIM), Maastricht University Medical Center+, Maastricht, the Netherlands; 8 Department of Haematology, University Medical Center Groningen, University of Groningen, Groningen, The Netherlands

**Keywords:** Venous thromboembolism, Embolism, Critical illness, Cohort studies, Critical care

## Abstract

**Background:**

The objective of this study was to describe the prevalence, incidence, prognostic factors, and outcomes of venous thromboembolism in critically ill patients receiving contemporary thrombosis prophylaxis.

**Methods:**

We conducted a pooled analysis of two prospective cohort studies. The outcomes of interest were in-hospital pulmonary embolism or lower extremity deep vein thrombosis (PE-LDVT), in-hospital nonleg deep vein thrombosis (NLDVT), and 90-day mortality. Multivariable logistic regression analysis was used to evaluate the association between predefined baseline prognostic factors and PE-LDVT or NLDVT. Cox regression analysis was used to evaluate the association between PE-LDVT or NLDVT and 90-day mortality.

**Results:**

A total of 2208 patients were included. The prevalence of any venous thromboembolism during 3 months before ICU admission was 3.6% (95% CI 2.8–4.4%). Out of 2166 patients, 47 (2.2%; 95% CI 1.6–2.9%) developed PE-LDVT and 38 patients (1.8%; 95% CI 1.2–2.4%) developed NLDVT. Renal replacement therapy (OR 3.5 95% CI 1.4–8.6), respiratory failure (OR 2.0; 95% CI 1.1–3.8), and previous VTE (OR 3.6; 95% CI 1.7–7.7) were associated with PE-LDVT. Central venous catheters (OR 5.4; 95% CI 1.7–17.8) and infection (OR 2.2; 95% CI 1.1–4.3) were associated with NLDVT. Occurrence of PE-LDVT but not NLDVT was associated with increased 90-day mortality (HR 2.7; 95% CI 1.6–4.6, respectively, 0.92; 95% CI 0.41–2.1).

**Conclusion:**

Thrombotic events are common in critically ill patients, both before and after ICU admittance. Development of PE-LDVT but not NLDVT was associated with increased mortality. Prognostic factors for developing PE-LDVT or NLDVT despite prophylaxis can be identified at ICU admission and may be used to select patients at higher risk in future randomized clinical trials.

***Trial registration*:**

NCT03773939.

## Background

Critically ill patients are at increased risk of developing venous thromboembolism (VTE), including both deep vein thrombosis (DVT) and pulmonary embolism (PE) [1, 2]. PE and lower extremity DVT (PE-LDVT) are often regarded as two manifestations of the same disease. Venous thrombosis may also occur at other sites, which has been referred to as nonleg deep vein thrombosis (NLDVT) [3–5]. Both PE-LDVT and NLDVT are associated with adverse outcomes [1–3, 6]. Thrombosis prophylaxis reduces the incidence of VTE and guidelines recommend pharmacologic prophylaxis for all critically ill patients if not contraindicated [7–9].

Despite prophylactic measures, thrombotic complications may still occur in critically ill patients. Two large randomized trials reported VTE incidences up to 6% in critically ill patients despite thrombosis prophylaxis [10, 11], while cohort studies reported incidences up to 37% [12–15]. NLDVT occurred in 2.2% of the patients in the PROTECT randomized trial [3]. Several prognostic factors for the development of VTE have been identified in critically ill patients, including central venous catheters, higher illness severity scores, renal failure, a history of VTE, higher body mass index, and vasopressor therapy [1, 13, 16]. The latter three were also recognized to be associated with failure of thrombosis prophylaxis in a post hoc analysis of the PROTECT trial [17].

Most studies on VTE incidence, prognostic factors, and outcomes are 5 to 10 years old, and methods and use of thrombosis prophylaxis as well as the overall level of care have improved over this period of time [7, 18]. Several of the previous studies excluded patients at risk of bleeding, which may have led to selection bias [10, 14, 17]. Most studies screened all patients for ‘asymptomatic’ thrombosis, although the clinical significance of these events remains to be established [19, 20].

More data on prognostic factors for failure of contemporary thrombosis prophylaxis in critically ill patients are needed to identify the target population for future high-quality randomized clinical trials. The objective of this study is to describe the prevalence, incidence, prognostic factors, and outcomes of both PE-LDVT and NLDVT in an unselected cohort of critically ill patients receiving routine thrombosis prophylaxis.

### Materials and methods

## Study design

This study is a pooled analysis of two prospective cohort studies: the Simple Intensive Care studies I (SICS-I) and the Simple Intensive Care studies II (SICS-II) including its pilot data [21, 22]*.* We performed the current analysis following a pre-published protocol (Clinicaltrials.gov, NCT03773939). Both the SICS-I and the SICS-II are single-center cohort studies that have been conducted in the University Medical Center Groningen. Inclusion criteria for both cohorts were the age of 18 years or older, unplanned Intensive Care Unit (ICU) admission, and an expected ICU stay of more than 24 h. Exclusion criteria were planned admission, inability or refusal to provide informed consent, and for the SICS-I also inability to include patients without interfering with clinical care (e.g., in case of continuous resuscitation efforts or mechanical circulatory support). The local institutional review board approved both cohort studies (2015/004 and 2018/203) and the current analysis (2019/078). Following national guidelines all patients received thrombosis prophylaxis with nadroparin 2850 IU once daily, provided there were no contraindications. In case of contraindications, patients received compression stockings. Pneumatic compression devices are not routinely used in our hospital. This study is reported according to the *Strengthening the Reporting of Observational Studies in Epidemiology* (STROBE) guideline [23].

### Data collection

Patient characteristics, such as demographical data, medication use, comorbidities, and severity of illness scores were registered at ICU admission. Candidate prognostic factors for the development of either PE-LDVT or NLDVT were defined a priori. These were either established prognostic factors for VTE or had been incorporated in previous VTE risk assessment models [24]. All factors were assessed once at ICU admission. We considered the following candidate predictors: active cancer, acute infection, acute renal failure, age, body mass index (BMI), cardiovascular failure, a central venous catheter (CVC), estrogen therapy, sex, limb paralysis, major surgery, mechanical ventilation, multiple trauma, previous VTE, respiratory failure, stroke, thrombophilic disorder, and vasopressor use. All candidate prognostic factors, including their a priori set definitions and units of measurement are listed in Additional file 1: Table S1. We applied the following post hoc changes to the protocol: age and BMI were assessed as continuous variables, renal replacement therapy was assessed as a separate predictor (in addition to renal failure), and reduced mobility was not assessed. All data had been prospectively collected within the SICS-II cohort, but some were not registered in SICS-I (CVC, estrogen use, major surgery, active cancer, multiple trauma, previous VTE, and thrombophilic disorder). The missing data for the patients included in the SICS-I cohort were manually retrieved from electronic health records.

### Outcome definitions

The outcomes of interest were in-hospital PE-LDVT, in-hospital NLDVT, and 90-day mortality. In-hospital PE-LDVT was defined as any objectively proven pulmonary embolism or lower extremity deep vein thrombosis during ICU or subsequent hospital stay. Lower extremity deep vein thrombosis was defined as acute thrombosis of the proximal lower-extremity veins (iliac, femoral, or popliteal), confirmed by compression ultrasonography, venography, CT, MRI, or autopsy. Pulmonary embolism was defined as acute thrombosis within the pulmonary vasculature as shown by ventilation-perfusion scan, CT angiography, or autopsy. Deep vein thrombosis in any other site was registered separately and referred to as NLDVT following previous reports [3]. NLDVTs included deep venous thrombosis in the jugular, subclavian, axillary, or brachial veins, but also thrombosis in the cava, mesenteric, portal, splenic, or renal veins. Superficial venous thrombotic events (i.e., cephalic or basilic vein thrombosis) and distal lower-extremity vein thromboses were not included under either of the two abovementioned outcome definitions. No screening protocol for the detection of PE-LDVT or NLDVT was used. Mortality was assessed at 90 days after ICU admittance based on the municipal record database.

### Statistical analysis

Data are presented as means with standard deviations (SD) or medians with interquartile ranges [IQR] depending on distributions. Effect estimates are presented as odds ratios (ORs) or hazards ratios (HRs) with 95% confidence intervals (CIs). We considered a two-sided *p* value < 0.05 statistically significant and performed no corrections for multiplicity. All analyses were performed using R software (R core team, 2019, version 3.6.0).

#### Prevalence, incidence, and anatomical location

We made a distinction between prevalent and incident events: any PE-LDVT or NLDVT diagnosed within 3 months before ICU admission was defined as a prevalent event, while events occurring after ICU admission were defined as incident. We registered the anatomical location for each event.

#### Missing data

Missing values were considered missing at random. Variables with less than 25% missing values were imputed with multiple imputations using chained equations using the *mice* package [25, 26]. The imputation model included baseline patient characteristics, all candidate prognostic factors, the Nelson–Aalen estimate of the cumulative hazard, and outcome variables. We created 30 imputed datasets with 20 iterations each. The imputed data were visually inspected and compared to observed data to assess the plausibility of imputations. Model convergence was monitored using iteration plots.

#### Models

Binary logistic regression analysis was conducted on each imputed dataset to determine baseline prognostic factors for the development of both PE-LDVT and NLDVT, including all a priori defined variables as candidate predictors (Additional file 1: Table S1). Patients with prevalent thrombotic events that had occurred within 24 h before ICU admission were excluded from all regression analyses because they had already achieved the outcome of interest. Univariable associations were assessed and a *p* value < 0.25 was used as threshold for inclusion in the multivariable models. A stepwise backward elimination method was used for constructing the multivariable models. Anticoagulant medication was entered in all multivariable models as a categorical variable (none, thrombosis prophylaxis, or therapeutic anticoagulation) because of its potential influence on the prognostic value of candidate factors. Parameter estimates were combined using Rubin’s rules at each stage of variable selection [27, 28]. Multicollinearity was assessed by analyzing variance inflation factors. We conducted two sensitivity analyses (S1 and S2) to assess the robustness of our findings. First, we reconstructed all logistic models on a complete case basis, excluding cases with missing data (S1). Second, we reconstructed all logistic models after excluding all PE-LDVTs or NLDVTs detected during the first two days of ICU admission, as these may represent prevalent instead of incident events (S2).

As a post hoc exploratory analysis, we used a multivariable Cox regression model on each imputed dataset to estimate the association between PE-LDVT or NLDVT, and 90-day mortality. The model was adjusted for the time-fixed covariates age, sex, admission type (medical, surgical, or other), APACHE IV score, and vasopressor therapy at ICU admission. PE-LDVT and NLDVT were included as time-dependent exposures to account for immortal time bias [29]. Parameter estimates were combined using Rubin’s rules.

#### Deviations from the protocol

The protocol was initially designed to describe the derivation and validation of a risk assessment model for the prediction of PE-LDVT in the ICU. The current study is a descriptive analysis of the main findings in the derivation cohorts (SICS-I and SICS-II). Recruitment for the external validation cohort is still ongoing. Decisions that were made on a post hoc basis in the current analysis have been marked as such in the preceding paragraphs.

## Results

### Study population

Recruitment occurred from 27 March 2015 to 22 July 2017 (SICS-I cohort) and 14 March 2018 to 10 July 2019 (SICS-II cohort). A total of 4987 patients were assessed for eligibility, of whom 2812 were eligible for inclusion, and 2208 were included (Additional file 1: Figure S1). Table 1 lists the baseline characteristics for this analysis (the two merged cohorts). Most patients were admitted for medical reasons, and 389 (17.6%) used therapeutic anticoagulant medication at home. The median hospital stay after ICU admission was 12 days (IQR 6–22). The number and frequency of missing values are provided in Additional file 1: Table S2*.*

**Table 1:** General characteristics at ICU admission

	*n* = 2208
Characteristics	
Age in years, mean (SD)	61.2 (15.1)
Sex, male, *n* (%)	1382 (62.6%)
BMI, mean (SD)	26.6 (5.4)
Admission type, *n* (%)^a^	
Medical	1426 (64.6%)
Acute surgery	679 (30.8%)
Other^b^	91 (4.1%)
Illness severity scores, mean (SD)	
APACHE II	20.6 (7.7)
APACHE IV	73.4 (30.2)
SAPS II	44.2 (17)
Comorbidities	
Chronic heart failure, *n* (%)	107 (4.8%)
Chronic renal impairment, *n* (%)	175 (7.9%)
Liver disease, *n* (%)	101 (4.6%)
Respiratory disease, *n* (%)	281 (12.7%)
Diabetes mellitus, *n* (%)	424 (19.2%)
Any previous venous thrombotic event, *n* (%)	220 (10%)
Venous thrombotic event in previous 3 months, *n* (%)	79 (3.6%)
Venous thrombotic event in previous 24 h, *n* (%)	42 (1.9%)
Therapeutic anticoagulation prior to current hospital admission, *n* (%)	389 (17.6%)

*APACHE* Acute Physiology And Chronic Health Evaluation, *BMI* body mass index, *ICU* Intensive Care Unit, *SAPS* Simplified Acute Physiology Score, *VTE* venous thromboembolism, *NLDVT* nonleg deep vein thrombosis

^a^Percentages do not add up to 100% due to rounding and missing data

^b^For example, unplanned admission due to complications after elective surgery

#### Prevalence of any thrombotic event before ICU admission

In the previous 3 months before ICU admission 79 patients had developed any thrombotic event accounting for a prevalence of 3.6% (95% CI 2.8–4.4%). Fifty-one of these patients (2.3%) had PE-LDVT and 28 (1.3%) NLDVT. In 42 of these patients (1.9%) the thrombotic event had occurred within 24 h before ICU admission, and these patients were excluded from further analyses.

#### Pulmonary embolism and lower extremity deep vein thrombosis

**Fig. 1:**
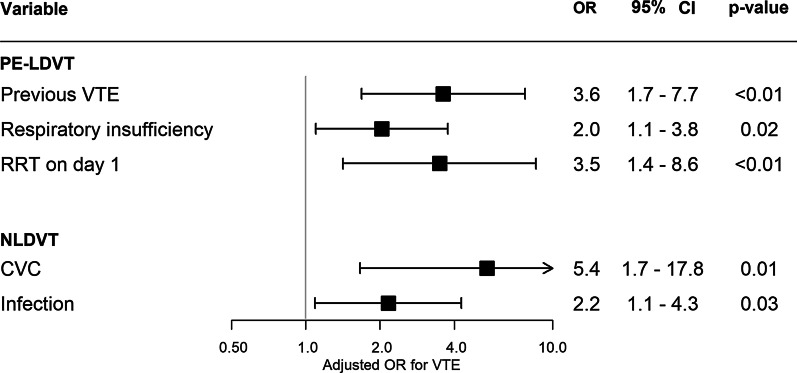
Prognostic factors for PE-LDVT and NLDVT: results from the main analysis. All analyses were adjusted for anticoagulant use in addition to the other variables. *CI* confidence interval, *CVC* central venous catheter, *NLDVT* nonleg deep vein thrombosis, *OR* odds ratio, *PE-LDVT* pulmonary embolism and lower extremity deep vein thrombosis, *RRT* renal replacement therapy, *VTE* venous thromboembolism

Out of 2166 patients, 47 (2.2%; 95% CI 1.6–2.9%) developed PE-LDVT either during ICU or subsequent hospital stay: DVT occurred in 15 patients and PE in 31 patients (Table 2). In one patient both DVT and PE were detected. The location of PE was subsegmental in 7 patients (22%), segmental in 18 patients (56%), central in 6 patients (19%), and unspecified in one (3%). Median time from ICU admission to development of PE-LDVT was 8.0 days (IQR 2.5–17.5). Results from univariable analyses are displayed in Additional file 1: Table S3. In multivariable analyses, renal replacement therapy (OR 3.5 95% CI 1.4–8.6; *p* < 0.01), respiratory failure (OR 2.0; 95% CI 1.1–3.8; *p* = 0.02), and a history of VTE (OR 3.6; 95% CI 1.7–7.7; *p* < 0.01) were statistically significant associated with the development of PE-LDVT (Fig. 1). Development of PE-LDVT was associated with increased risk of 90-day mortality (HR 2.7; 95% CI 1.6–4.6; *p* < 0.01).

**Table 2:** Prognostic factors, anticoagulant medication, and outcomes

	No VTE	PE-LDVT	NLDVT
	*n* = 2081	*n* = 47	*n* = 38
Prognostic factors at ICU admission			
Active cancer, *n* (%)	317 (15.2%)	10 (21.3%)	10 (26.3%)
Acute infection, *n* (%)	397 (19.1%)	15 (31.9%)	14 (36.8%)
Acute renal failure stage, *n* (%)^a^			
AKI 1	329 (15.8%)	3 (6.4%)	10 (26.3%)
AKI 2	238 (11.4%)	6 (12.8%)	4 (10.5%)
AKI 3^b^	229 (11%)	11 (23.4%)	9 (23.7%)
Renal replacement therapy	74 (3.6%)	6 (12.8%)	5 (13.2%)
Age in years, mean (SD)	61.4 (15.1)	59.4 (13.4)	58.9 (15)
BMI, mean (SD)	26.6 (5.4)	25.8 (4.5)	27.7 (5.2)
Cardiovascular failure, *n* (%)	426 (20.5%)	6 (12.8%)	5 (13.2%)
Central venous access, *n* (%)	1327 (63.8%)	38 (80.9%)	35 (92.1%)
Estrogen therapy, *n* (%)	20 (1%)	0 (0%)	0 (0%)
Limb paralysis, *n* (%)	169 (8.1%)	1 (2.1%)	1 (2.6%)
Major surgery, *n* (%)	789 (37.9%)	23 (48.9%)	17 (44.7%)
Mechanical ventilation, *n* (%)	1448 (69.6%)	36 (76.6%)	32 (84.2%)
Multiple trauma, *n* (%)	193 (9.3%)	5 (10.6%)	0 (0%)
History of VTE, *n* (%)	167 (8%)	10 (21.3%)	1 (2.6%)
Respiratory failure, *n* (%)	429 (20.6%)	17 (36.2%)	9 (23.7%)
Sex, male, *n* (%)	1301 (62.5%)	28 (59.6%)	28 (73.7%)
Stroke, *n* (%)	176 (8.5%)	1 (2.1%)	0 (0%)
Thrombophilic disorder, *n* (%)	15 (0.7%)	1 (2.1%)	0 (0%)
Vasopressor use, *n* (%)	1086 (52.2%)	28 (59.6%)	23 (60.5%)
Anticoagulant medication at ICU admission			
Antiplatelet medication, *n* (%)	591 (28.4%)	13 (27.7%)	5 (13.2%)
Anticoagulant medication, *n* (%)^a^			
None	380 (18.3%)	5 (10.6%)	3 (7.9%)
Thrombosis prophylaxis	1227 (59%)	34 (72.3%)	30 (78.9%)
Therapeutic anticoagulation/other	462 (22.2%)	8 (17%)	5 (13.2%)
Outcomes			
ICU length of stay in days, median (IQR)	2.8 (1.6–5.7)	4.9 (2.3–15.6)	5.3 (2.6–15)
Hospital length of stay in days, median (IQR)^c^	12 (6–22)	23 (14.2–45.5)	28 (15.2–59.8)
In-hospital mortality, *n* (%)	453 (21.8%)	11 (23.4%)	7 (18.4%)
90-day mortality, *n* (%)	564 (27.1%)	15 (31.9%)	7 (18.4%)

*BMI* body mass index, *ICU* intensive care unit, *n* number, *NLDVT* nonleg deep vein thrombosis, *PE-LDVT* pulmonary embolism and lower extremity deep vein thrombosis, *VTE* venous thromboembolism

^a^Percentages may not add up to 100% due to rounding and/or missing data.

^b^Includes renal replacement therapy

^c^Starting from date of ICU admittance

#### Nonleg deep vein thrombosis

A total of 38 patients (1.8%; 95% CI 1.2–2.4%) developed NLDVT. Nineteen patients (50%) developed thrombosis of the upper-extremity veins, 13 (34%) developed portal vein thrombosis and 6 (16%) developed NLDVT in another location (e.g., in mesenteric, renal or splenic veins). Median time from ICU admission to the development of NLDVT was 8.5 days (IQR 1.5–21). Results from univariable analyses are displayed in Additional file 1: Table S3. In multivariable analyses, CVC (OR 5.4; 95% CI 1.7–17.8; *p* = 0.01) and infection (OR 2.2; 95% CI 1.1–4.3; *p* = 0.03) were statistically significant associated with the development of NLDVT (Fig. 1). There was no statistically significant association between NLDVT and 90-day mortality (HR 0.92; 95% CI 0.41–2.1; *p* = 0.85). No patients developed both PE-LDVT and NLDVT.

#### Sensitivity analyses

**Fig. 2:**
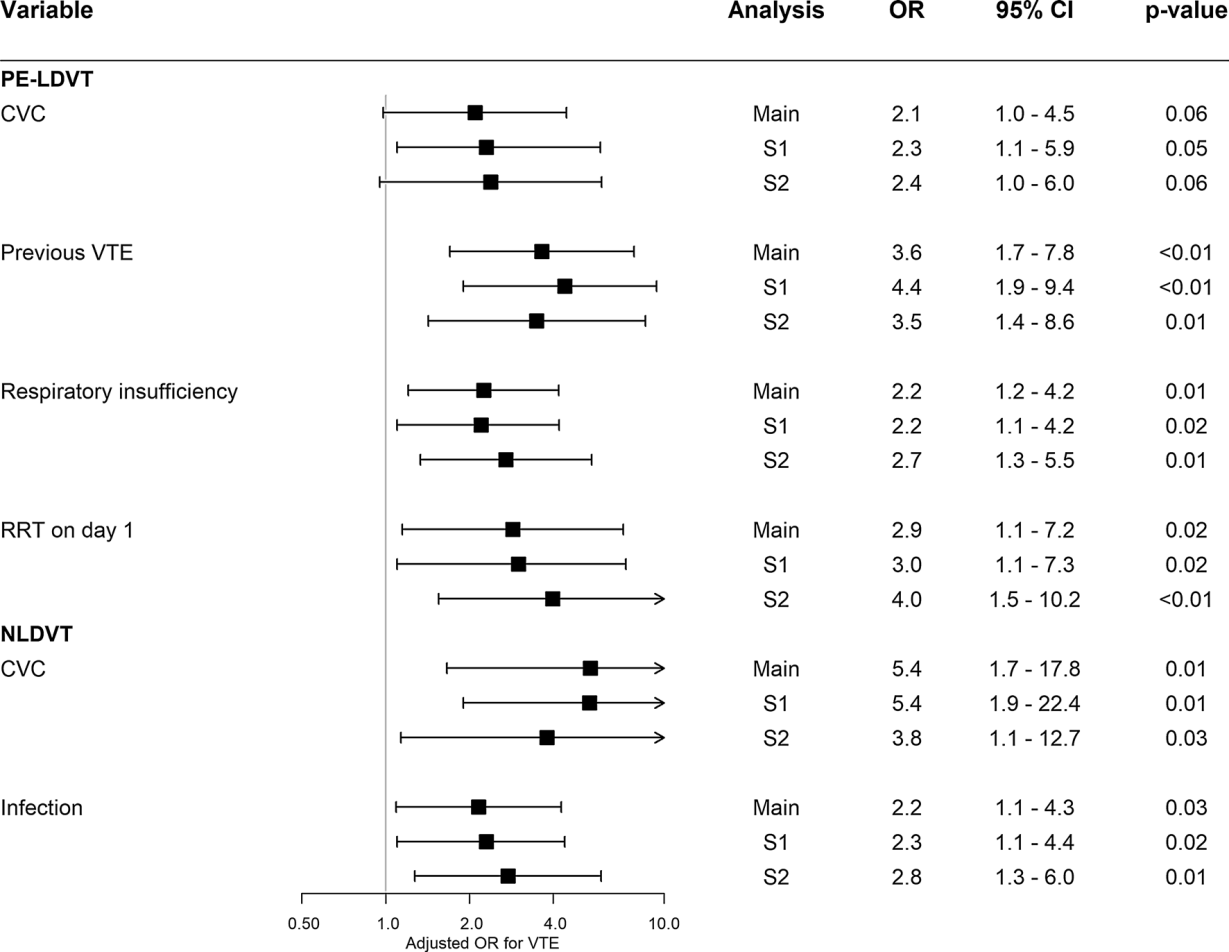
Prognostic factors for PE-LDVT and NLDVT: results from the main analysis and sensitivity analyses. All analyses were adjusted for anticoagulant use in addition to the other variables. Main: main analysis including CVC as a predictor, results, therefore, differ slightly from the text and Fig. 1; S1: sensitivity analysis 1, the complete case analysis; S2: sensitivity analysis 2, excluding patients with outcome events detected within the first two days of ICU admission. *CI* confidence interval, *CVC* central venous catheter, *NLDVT* nonleg deep vein thrombosis, *OR* odds ratio, *PE-LDVT* pulmonary embolism and lower extremity deep vein thrombosis, *RRT* renal replacement therapy, *VTE* venous thromboembolism

In the complete case analysis (S1), the association between CVC presence and PE-LDVT development was statistically significant, in contrast to the main analysis (Fig. 2). For all other prognostic factors, the complete case analysis confirmed the main analysis. Results from the sensitivity analysis excluding all patients with outcome events during the first two days after ICU admission (S2) were fully concurrent with the main analysis (Fig. 2).

## Discussion

In this analysis of two prospective cohorts of acutely admitted critically ill patients, we observed 2.2% incident PE-LDVT and 1.8% NLDVT after ICU admission. The prevalence of any thrombotic event 90 days before ICU admission was 3.6%. Since we did not employ any systematic screening, these data reflect VTEs that were detected due to clinical signs or symptoms that prompted further diagnostic evaluation. Development of PE-LDVT but not NLDVT was associated with increased 90-day mortality in a multivariable cox regression model adjusted for several confounding factors. Baseline prognostic factors for PE-LDVT development were renal replacement therapy, admittance for respiratory failure, and a history of VTE; while baseline prognostic factors for NLDVT were CVC presence and admittance for acute infection. A high proportion of patients received antiplatelet, prophylactic, or therapeutic anticoagulation at the time of their ICU admission, and interestingly, despite the use of therapeutic anticoagulant medication at the time of ICU admission, several patients still developed venous thromboembolism. We did not register details on therapeutic anticoagulant medication use at the time of PE-LDVT development, and it is possible therapy was temporarily halted due to invasive procedures. Our data on prognostic factors for development of thrombosis despite routine prophylactic strategies can be used by clinicians for crude risk assessment, and may be used to select patients at high risk of thrombotic complications in future randomized clinical trials.

We did not include critically ill patients with coronavirus disease 2019 (COVID-19) since the SICS-I and SICS-II studies were conceived before the pandemic. The incidence of VTE in COVID-19 is high, consistent with previous reports on other viral pneumonias, for example as caused by H1N1 [30, 31]. Results from a study on incidence and prognostic factors of VTE in unselected critically ill patients would be heavily influenced if a large proportion of included patients suffered from a single condition associated with high risk of VTE. Therefore, we believe that as the burden of the pandemic ultimately lowers, and the proportion of critically ill patients with COVID-19 ‘normalizes,’ our results and conclusions should remain valid.

The incidence of thrombotic events in this study reflects daily clinical practice, and is lower than most previous studies that have reported incidences up to 37% [10, 11, 13–15]. In contrast, the prevalence of VTE before ICU admittance was high, which may be a reflection of baseline comorbidities. Several factors may account for the observed VTE incidence. We limited follow-up to the in-hospital period which may have led to an underestimation as previous studies have shown up to a third of events may occur weeks after discharge [32]. Also, mortality is a competing risk for VTE and may have precluded development of symptomatic events. In contrast, many of the previous studies applied stringent selection criteria, focused on particular very ill subgroups, or employed VTE screening methods, all of which in turn may have contributed to detecting a higher VTE incidence. Whether early detection and treatment of ‘asymptomatic’ VTEs through screening will improve the overall prognosis of critically ill patients still needs to be established. Previous data suggested a poor relationship between asymptomatic and symptomatic events in thromboprophylaxis trials, suggesting not all asymptomatic VTE will eventually evolve to become symptomatic [19]. Nevertheless, traditional signs and symptoms of VTE may be unreliable in critically ill patients, prohibiting a symptom-based dichotomization. Recently, in a pre-planned subgroup analysis from the PREVENT trial, Arabi and colleagues found a beneficial effect of VTE surveillance on 90-day mortality, which suggests that asymptomatic events do contribute to poor outcomes [20]. Only a well-designed randomized trial will be able to elucidate this question. Such a trial could focus on early VTE detection or, alternatively, treat high-risk patients with a higher dose of thrombosis prophylaxis.

Prognostic factors and outcomes of PE-LDVT were different from those of NLDVT, reflecting that these events should be regarded as separate entities [4]. Occurrence of PE-LDVT and not NLDVT was associated with increased 90-day mortality. This is in line with recent cohort studies in which critically ill patients who developed PE-LDVT (or a comparable composite) had higher mortality rates, while NLDVT was not associated with increased mortality [3, 15, 16]. Our study also replicates results from earlier studies that used surveillance ultrasound to detect VTE, confirming the value of previous VTE and renal failure as a prognostic factors for PE-LDVT in critically ill patients [1, 13, 17]. In contrast, respiratory failure was not previously identified as a prognostic factor, while sex, BMI, and vasopressor use could not be confirmed to be associated with PE-LDVT. Prognostic factors for NLDVT were CVC presence and acute infection. The first finding is not surprising, given that CVCs are a well-known risk factor for thrombosis [2, 33]. The latter is a new finding that needs confirmation in a follow-up study. In contrast to previous results, malignancy was not associated with NLDVT [3].

Our study has several strengths and limitations. One of its main strengths is the prospective design with high quality data collection and a priori defined candidate prognostic factors to assess both PE-LDVT and NLDVT in an unselected critically ill population receiving contemporary thrombosis prophylaxis. We applied statistical methods to handle missing data and performed sensitivity analyses to assess our findings’ robustness. Nevertheless, several limitations should be recognized. First, although the inclusion criteria were broad, the single center design limits the external validity of our findings and we encourage replication in an independent sample. Second, we did not collect data on non-pharmacological prophylactic interventions, such as compression stockings or mechanical compression devices, and were therefore unable to acknowledge for these in the analyses. Third, follow-up was limited to the hospital stay, which may have led to underestimating the PE-LDVT incidence as previous studies have estimated that up to one third of all events may occur after hospital discharge [32]. Relying on symptomatic events may have further contributed to a lower VTE incidence, although systematic screening may in turn lead to overdiagnosis. Fourth, we did not collect data on time-dependent prognostic factors that may have arisen during ICU admission. Fifth, the incidence of PE-LDVT and NLDVT was lower than anticipated, which increases the risk of model overfitting and led to wide confidence intervals. Finally, several events were detected shortly after ICU admission, and may represent prevalent instead of incident events. Still, excluding these events in a sensitivity analysis did not substantially impact the results.

## Conclusions

In conclusion, thrombotic events are common in critically ill patients, both before and after ICU admittance. Development of PE-LDVT but not NLDVT was associated with increased mortality. Prognostic factors for developing PE-LDVT or NLDVT despite prophylaxis can be identified at ICU admission and may be used to select patients at higher risk in future randomized clinical trials.

## Supplementary Information

**Additional file 1**. **Table S1**. Prognostic factor definitions. **Table S2**. Missing data. **Table S3**. Prognostic factors for PE-LDVT and NLDVT: univariable analyses. **Figure S1**. Flow-chart of patient inclusion.

## Data Availability

The datasets used and/or analyzed during the current study are available from the corresponding author on reasonable request.

## Abbreviations

APACHE IV: Acute Physiology and Chronic Health Evaluation
BMI: Body mass index
CVC: Central venous catheter
DVT: Deep vein thrombosis
ICU: Intensive care unit
IQR: Interquartile range
NLDVT: Nonleg deep vein thrombosis
OR: Odds ratio
PE: Pulmonary embolism
PE-LDVT: Pulmonary embolism or lower extremity deep vein thrombosis
SD: Standard deviation
SICS: Simple Intensive Care Studies
