# Supplementary material for: Incidence, prognostic factors, and outcomes of venous thromboembolism in critically ill patients: data from two prospective cohort studies
Source: Crit Care. 2021 Jan 12;25:27. doi: 10.1186/s13054-021-03457-0 (PMC7801861; doi:10.1186/s13054-021-03457-0)
Supplement: Supplementary file 1 — Additional file 1. Table S1. Prognostic factor definitions. Table S2. Missing data. Table S3. Prognostic factors for PE-LDVT and NLDVT: univariable analyses. Figure S1. Flow-chart of patient inclusion. [file 13054_2021_3457_MOESM1_ESM.docx]

Additional information to

**Incidence, prognostic factors, and outcomes of venous thromboembolism in critically ill patients: data from two prospective cohort studies**

Ruben J. Eck, MD; Lisa Hulshof, MD; Renske Wiersema, PhD; Chris H.L. Thio, PhD; Bart Hiemstra, MD, PhD; Niels C. Gritters van den Oever, MD; Reinold O.B. Gans, MD, PhD; Iwan C.C. van der Horst, MD, PhD; Karina Meijer, MD, PhD; Frederik Keus, MD, PhD; SICS Study Group

**Contents**

[**Table S1.** Prognostic factor definitions 2](#_Toc55235682)

[**Figure S1.** Flow-chart of patient inclusion 3](#_Toc55235683)

[**Table S2.** Missing data 3](#_Toc55235684)

[**Table S3.** Prognostic factors for PE-LDVT and NLDVT: univariable analyses 4](#_Toc55235685)

| **Table S1.** Prognostic factor definitions | |
| --- | --- |
| **Prognostic factor** | **Definition** |
| Active cancer | Cancer diagnosed within the previous 6 months; recurrent, regionally advanced or metastatic cancer; cancer for which treatment had been administered within 6 months before ICU admission; or hematologic cancer that is not in complete remission. Non-melanoma skin cancer will be excluded. |
| Acute infection | Proven infection at ICU admission or within 24 hours of admission. Defined as either positive culture results or gram staining, peroperative findings (ie feces during laparotomy) or strong suspicion based on radiology in combination with clinical findings. |
| Acute renal failure or renal replacement therapy | Acute renal injury within 24 hours of ICU admission according to KDIGO criteria stage 2 or 3, or renal replacement therapy within 24 hours of ICU admission |
| Age | Years |
| BMI | kg/m2 |
| Cardiovascular failure | Cardiopulmonary resuscitation and/or dysrhythmia before ICU admission |
| CVC | Central venous line at ICU admission or within 24 hours of admission |
| Estrogen therapy | Exogenous estrogen |
| Limb paralysis | Hemiparesis, hemiplegia, paraplegia, or quadriplegia |
| Sex | Sex |
| Major surgery | Every extensive surgical procedure (>45 min) that either: involves vital organs; is hazardous to life; or involves invasive orthopedic surgery on joints or bones, during hospital admission or within the previous month of ICU admission |
| Mechanical ventilation | Mechanical ventilation at ICU admission or within 24 hours of admission |
| Multiple trauma | Any multiple trauma during hospital admission or within the previous month of ICU admission |
| Previous VTE | Documented VTE in medical history |
| Respiratory failure | According to APACHE II or APACHE IV admission diagnosis |
| Stroke | Cerebral embolus, occlusion, bleeding or infarction. Diagnosis in 24 hours before, or first hour of ICU admission |
| Thrombophilic disorder | Documented deficiency of antithrombin, protein C or S, carriage of factor V Leiden or G20210A prothrombin polymorphism, antiphospholipid syndrome and/or lupus anticoagulant |
| Vasopressor use | Use of any inotrope or vasopressor at ICU admission or within 24 hours of admission |
| *BMI: body mass index; CVC: central venous catheter; VTE: venous thromboembolism* | |

### **Figure S1.** Flow-chart of patient inclusion


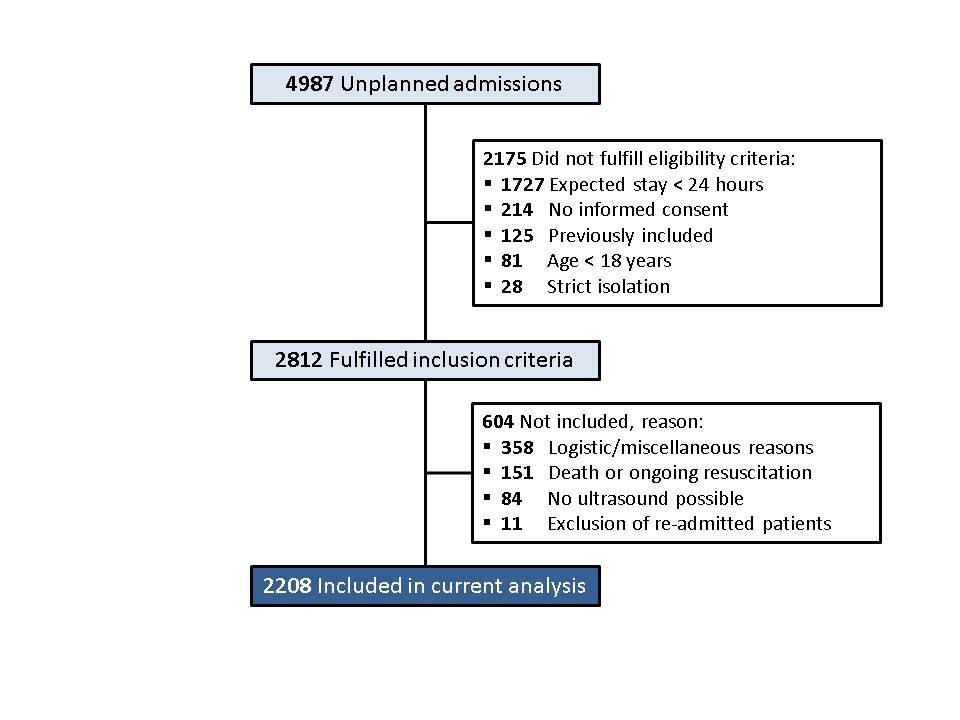


| **Table S2.** Missing data | | |
| --- | --- | --- |
| **Variable** | **No. complete data (%)** | **No. missing data (%)** |
| Apache II score | 1969 (89.2%) | 239 (10.8%) |
| Apache IV score | 1936 (87.7%) | 272 (12.3%) |
| AKI stage | 2075 (94%) | 133 (6.0%) |
| BMI | 2171 (98.3%) | 37 (1.7%) |
| CVC | 2111 (95.6%) | 97 (4.4%) |
| *Only variables with missingness of > 1% are displayed; APACHE: acute physiology and chronic health evaluation; BMI: body mass index; CVC: central venous catheter* | | |

### **Table S3.** Prognostic factors for PE-LDVT and NLDVT: univariable analyses

|  | **PE-LDVT** | | **NLDVT** | |
| --- | --- | --- | --- | --- |
| **Variable** | **Odds ratio (95% CI)** | **P-value** | **Odds ratio (95% CI)** | **P-value** |
| Active cancer | 1.5 (0.7 – 3.0) | 0.28 | 2.0 (0.95 – 4.1) | 0.07 |
| Acute infection | 1.9 (1.0 – 3.6) | 0.04 | 2.5 (1.3 – 4.9) | < 0.01 |
| Acute renal failure |  |  |  |  |
| None | Reference |  | Reference |  |
| Stage 1 | 0.5 (0.2 – 1.7) | 0.25 | 2.4 (1.04 – 5.3) | 0.04 |
| Stage 2 | 1.2 (0.5 – 3.0) | 0.65 | 1.3 (0.4 – 3.9) | 0.66 |
| Stage 3 | 2.1 (1.0 – 4.4) | 0.04 | 2.9 (1.3 – 6.7) | 0.01 |
| Renal replacement therapy | 3.8 (1.6 – 9.2) | < 0.01 | 3.9 (1.5 – 10.2) | < 0.01 |
| Age | 0.99 (0.97 – 1.01) | 0.39 | 0.99 (0.97 – 1.01) | 0.33 |
| BMI | 0.97 (0.9 – 1.03) | 0.32 | 1.03 (0.98 – 1.09) | 0.20 |
| Cardiovascular failure | 0.6 (0.2 – 1.4) | 0.20 | 0.6 (0.2 – 1.6) | 0.31 |
| CVC | 2.1 (1.0 – 4.4) | 0.04 | 5.9 (1.8 – 19.2) | < 0.01 |
| Estrogen therapy | Not estimated ^a^ |  | Not estimated ^a^ |  |
| Limb paralysis | 0.3 (0.03 – 1.8) | 0.17 | 0.3 (0.04 – 2.3) | 0.25 |
| Sex (male) | 0.9 (0.5 – 1.6) | 0.66 | 1.7 (0.8 – 3.5) | 0.16 |
| Major surgery | 1.6 (0.9 – 2.8) | 0.13 | 1.3 (0.7 – 2.5) | 0.41 |
| Mechanical ventilation | 1.4 (0.7 – 2.8) | 0.34 | 2.8 (1.1 – 7.3) | 0.03 |
| Multiple trauma | 1.2 (0.5 – 3.0) | 0.72 | Not estimated ^a^ |  |
| Previous VTE | 3.1 (1.5 – 6.4) | < 0.01 | 0.3 (0.04 – 2.2) | 0.23 |
| Respiratory failure | 2.2 (1.2 – 4.0) | 0.01 | 1.2 (0.6 – 2.5) | 0.68 |
| Stroke | 0.2 (0.03 – 1.8) | 0.16 | Not estimated ^a^ |  |
| Thrombophilic disorder | 3.1 (0.4 – 23.6) | 0.29 | Not estimated ^a^ |  |
| Vasopressor use | 1.3 (0.7 – 2.4) | 0.33 | 1.4 (0.7 – 2.7) | 0.32 |
| *^a^ Not estimated because of (quasi-)complete separation, most likely due to low number of cases; BMI: body mass index; CI: confidence interval; CVC: central venous catheter; NLDVT: nonleg deep vein thrombosis; PE-LDVT: Pulmonary embolism or lower extremity deep vein thrombosis; VTE: venous thromboembolism.* | | | | |
